# Protein Analysis-on-Chip Systems in Foodomics

**DOI:** 10.3390/nu4101475

**Published:** 2012-10-16

**Authors:** Filomena Nazzaro, Pierangelo Orlando, Florinda Fratianni, Aldo Di Luccia, Raffaele Coppola

**Affiliations:** 1 Institute of Food Science, ISA-CNR, Via Roma 64, Avellino 83100, Italy; Email: fratianni@isa.cnr.it (F.F.); a.diluccia@unifg.it (A.D.L.); direttore@isa.cnr.it (R.C.); 2 Institute of Protein Biochemistry, IBP-CNR, Via P. Castellino 121, Napoli 80124, Italy; Email: p.orlando@ibp.cnr.it; 3 Department of Food Science, University of Foggia, Via Napoli 25, Foggia 71100, Italy

**Keywords:** lab-on-chip, protein, microisoelectrofocusing, proteomic, food

## Abstract

Protein compositional data can address nutritional, packaging, origin/authenticity, processing history, safety and other quality questions. Such data has been time-consuming and expensive to generate until recently but “protein analysis on a chip” systems are now available that can analyze a complex food sample in a few minutes and do not require great protein analytical expertise. We review some of the main new approaches with examples of their application and discuss their advantages and disadvantages.

## 1. Protein Analysis and Lab-on-Chip Method

*Highlights: *Faster and finer approaches for the analysis of food proteins are necessary for the assurance of food quality and safety, as well as for consumer health; lab-on-chip electrophoresis tries to overcome the difficulties of protein analysis, offering a micro-platform for a quick analysis of all proteins present in a food matrix beyond the confines of a traditional laboratory.

**Figure 1:**
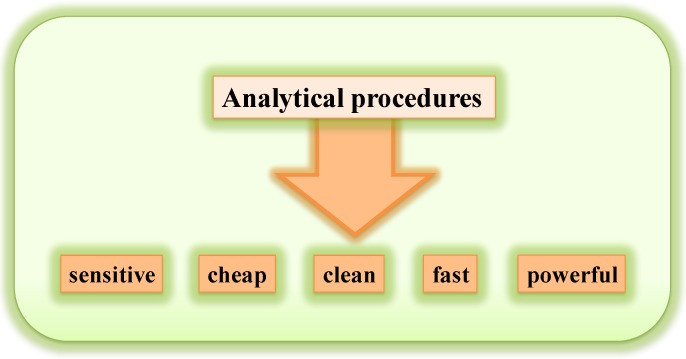
Some of the most important requisites for the modern analytical techniques.

Proteins are one of the fundamental constituents in many varieties of foods. Thus, separation and quantification of proteins can represent a key step in the evaluation of food nutrition and quality. Chromatography and electrophoresis, as proteomic tools, have proven to be useful in the identification of food components. However, these methods are generally slow and confined to the laboratory, because of their complexity, their back-up resources and the trained operation required for their use. SDS PAGE (Sodium Dodecyl Sulphate Polyacrylamide Gel Electrophoresis) under reducing conditions is still one of the most important techniques used in all laboratories for the separation and sizing of proteins. The necessary steps of this method involve preparing and assembling a gel, mixing a protein sample with a buffer containing SDS while warming the mixture in boiling water, loading the protein-SDS mixture into wells of the gel and performing the electrophoresis. After running, gels are fixed, stained/de-stained, and separated proteins can be quantified by image analysis with specific equipment. Generally, even performed on a mini-scale, this gel method requires several hours for its conclusion and gives detailed information essentially on molecular size and relative abundance of proteins; furthermore, it requires the use of high volumes of materials such as buffers and solvents, many of which are expensive and toxic for humans and the environment. Faster and better analysis of food proteins leads to improvement and implementation of applications in the assurance of food quality and safety, consumer health, as well as an economic, religious or social impact. This is particularly relevant, for example, when it is necessary to have rapid information about the presence of allergens in foodstuff such as infant formulas, arising from improper manufacturing practice, or to detect the presence of proteins indicating a fraudulent use of heat-treated milk instead of raw milk in the manufacturing of precious typical cheeses, or even when some products are manufactured for people with precise religious restrictions for food (e.g., banning of pork meat for Muslims, banning of fermented food for Jewish people). Today, to overcome the difficulties of protein analysis related to time, sensitivity and cost, new technologies, like microtechnologies, can support the requirement of scientific and legislative fields by combining an increased speed of analysis and results with improved sensitivity of methods [1,2] (Figure 1). 

**Figure 2:**
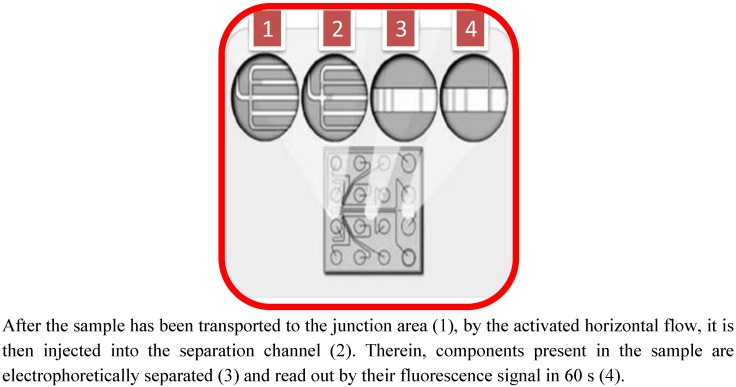
Microfluidic realization of capillary electrophoresis analysis on the electrokinetic platform.

Microfabricated (or microchip) devices have been developed to perform and integrate multiple analytical processes, called “lab-on-chip”, on a unique chip platform, including sample pre-treatment, solution distribution/mixing, separation, detection, *etc.* [3,4,5]. In recent years, lab-on-chip electrophoresis has tried to overcome the difficulties of protein analysis, with regard to time, sensitivity and cost, thereby offering a platform for quickly analyzing all proteins present in a food matrix beyond the confines of a traditional laboratory. Due to mainly the high separation efficiency, electrophoresis of proteins on a microchip is generally fast, typically taking place in a few seconds to a few minutes. Yao *et al.* [6] were the first to perform SDS-PAGE in a microfabricated channel, and the separations were completed in less than one minute. By coupling an on-chip dye staining with an electrophoretic dilution step (similar to a destaining step), Bousse *et al.* [7] obtained outstanding resolutions for microchip electrophoresis of proteins. Han and Singh [8] and Herr and Singh [9] utilized an in-channel photopolymerization approach to prepare polyacrylamide gels inside a microchip channel for gradient SDS-PAGE and achieved a separation faster than 30 s *per* run [10]. Tsai *et al.* [11] tested simultaneous separations of both native and SDS-denatured proteins on a single microchip with 36 microchannels. More recently, He and Herr developed a microfluidic system for protein immunoblotting [12,13], that is an appropriate method in protein identification specifically in the study of food proteins from complex animal and vegetable matrices. The success of the electrophoresis on a microchip is mainly related to the small sample volume needed for the protein separation. The time for run and analysis for each sample of protein ranges between one and three minutes and, for each run, the system allows processing of very small volumes of materials, typically less than 0.5 mL total volume per chip, including the sample (generally max. 10 μL) and reagents. This aspect can be considered very attractive, as very low levels of reagents are used and little waste is generated, and minimal sample volumes are required. The lab-on-chip system uses the principle of capillary electrophoresis for analyzing protein composition. The “heart” of the equipment is the chip, generally about 5 cm square, in which micro-wells are filled with ten samples plus reagent. Depending on the chip selected, the technique is capable of separating proteins up to 260 kDa and also claims to have a linear dynamic range of 2.5–1000 μg/μL. Innovations in protein separation by electrophoresis have seen the implementation of such a technique: The area of microfluidic systems is a quickly developing field and, as for genetics, the literature offers papers and reviews devoted to microfluidic chips for protein analysis [14,15,16,17]. Many of the microchip electrophoresis systems separate, in a miniaturized way, proteins according to their mass. Such systems include a detection part with one or two fixed wavelengths, and a chamber to place the chip after being filled with sieved polymers and protein samples. In these systems, protein sizing is obtained by capillary gel electrophoresis, with denatured protein-SDS complexes [7]. Several companies have launched full microfluidic systems or platforms including the detection system, power supplier and all that is required to perform electrophoresis separation on a chip. A typical modular design integrates a fluidic microseparation chip, lasers, optics, a high-voltage power supply, electronic controls, data algorithm and a user interface. The principle of electrophoresis on a chip is very similar to a SDS PAGE. Samples are heat denatured in a high concentration of SDS, which coats the protein, resulting in a net negative protein surface charge that enables electrophoretic separation. The protein chip is prepared by pressure priming the microfluidic channels with gel-dye (which serves as both a sieving matrix for the separation of the proteins and a staining solution) and destaining solutions. After priming, a marker solution is pipetted onto the chip. A microfraction of each sample is aspirated by vacuum through a capillary sipper and into the microfluidic channels of the chip; during this step, the sample is diluted with a marker solution, charged in one reference well, which is subsequently used as a reference for migration time and determination of the relative concentration of the samples. Protein destaining is accomplished using a dilution step achieved by electrokinetically flowing SDS-free ions into the separation channel at the destain intersection. This causes the dye-SDS-protein fluid stream to focus. In a few milliseconds, diffusion of free SDS micelles into the SDS-free fluid leads to the breakup of the micelles and a significant drop in the background fluorescence. SDS micelles bound to the protein remain intact. Since the proteins are still coated with SDS-dye and retain their fluorescence, the separated protein bands are detected downstream of the dilution point by using laser induced fluorescence. Free solution dye molecules are not detected because they are only fluorescent in the hydrophobic environment of the SDS micelles. [17]. The basis of protein analysis on chip is shown in Figure 2.

At present, use of a microfluidic chip for protein analysis is considered without doubt to be a system with high and fast resolution and reproducibility also when compared to conventional methods, such as PAGE, Reversed Phase-High Performance Liquid Chromatography (RP-HPLC), and to the conventional Capillary Electrophoresis (CE) [18,19] (Table 1). 

**Table 1:** Comparison of some routine methods of food protein analysis. Adapted from [18].

Factors considered	PAGE	RP-HPLC	Conventional CE	Microfluidic CE
**Time for setting gel or regenerating column**	60 min	10 min	2–3 min	2–3 min
**Sample extraction**	depending on source	depending on source	depending on source	depending on source
**Run time**	from 30 to 240 min	from 10 to 90 min	10 min	1–3 min
**Visualization of proteins**	from 2 h to overnight	instant	instant	instant
**Health risks for operator**	moderate	low	low–medium	low
**Cost of equipment**	low	high	high	medium
**Cost of consumables**	low	medium	medium	low–medium

**Figure 3:**
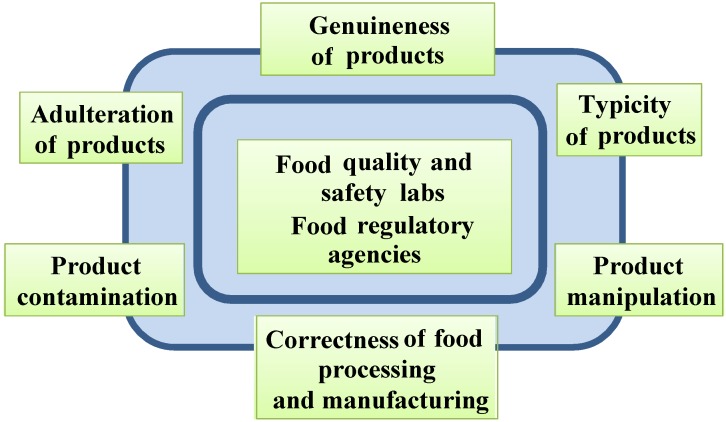
Scheme of the most important interactions of laboratories and regulatory agencies in food science.

Generally, the most recent applications of microchip electrophoresis for the analysis of proteins in food include the characterization of protein extracts [20], which are capable of giving information about [21,22]; or monitoring and optimizing the technological process [23] or the quality of a protein extract [24,25]; or detecting high-quality value products adulterated with products of inferior quality [26]. Examination of food quality and safety is a critical task in analytical chemistry, as it concurrently represents an important way to preserve human health (Figure 3). 

Use of microchip analysis for the identification of wheat varieties and for prediction of flour quality has been described by Uthayakumaran *et al.* [27,28], who analyzed about 40 commonly grown Australian wheat varieties, obtaining a complete monodimensional profile of ten samples, including extraction of polypeptides, centrifugation and electrophoresis on chip, in less than 1 h. This gave details on the high and low molecular weight glutenin subunits, which are important parameters for the prediction of the potential of breeding lines for dough quality. Such analysis could be complementary not only for providing complete information about both varieties and likely processing properties, but also to reflect on the contributions of growth/storage conditions on the quality of wheat and flour [29,30]. In another study, unlabeled proteins (lysozyme, conalbumin and ovalbumin) from a diluted real sample of chicken egg white [31] were separated under native conditions in less than 2 min by microchip electrophoresis using fused silica chips and a deep UV-fluorescence detection. More recently, the whey protein fractions from the milk of 120 Mediterranean water buffalo individual were analyzed by microchip electrophoresis: This technique allowed very fast separation of the major whey proteins as the run time was about 3 min per chip well. The resolution achieved by this method enabled the complete separation of the three major whey proteins [32] and, as for the other cases, made microchip electrophoresis a convenient alternative to SDS-PAGE [33] with considerably higher throughput, so that the instrumental response showed nearly linear behavior, leading to average *r*^2^ coefficients of 0.95 for α-lactalbumin, 0.94 for β-lactoglobulin and 0.93 for serum albumin over all the chip runs. The lab-on-chip method was used by Butikofer *et al.* [34], for the determination of the percentage of α-lactalbumin + β-lactoglobulin of total protein, in raw, pasteurized and UHT treated milk, comparing such analysis with conventional methods capable of quantifying the total amount of whey proteins in relation to casein in heat treated milk, such as capillary electrophoresis with UV detection. Both methods were capable of measuring the percentage of proteins considered and to separate α-lactalbumin and β-lactoglobulin. Therefore they could be used to detect alteration of the natural proportion of the two whey proteins resulting from new or incorrect technological processes. The determination of the percentage of α-lactalbumin and β-lactoglobulin by microchip PAGE was considered by the authors to be more favorable, because of the faster protein separation and the lower acquisition costs compared to conventional capillary electrophoresis. The technique was applied to the separation and quantification of different milk proteins [35,36] and compared with the traditional SDS-PAGE. The method separated all major milk proteins when standard protein solutions were used. In the milk system, α-lactalbumin, β-lactoglobulin, αs-casein, β-casein and κ-casein were readily separated, and the resolution was comparable with SDS-PAGE. However, Anema [35] highlighted a sub-optimal resolution of the immunoglobulins, lactoferrin and bovine serum albumin from the background in the microfluidic chip technique, compared to SDS-PAGE. Lab-on-chip electrophoresis provided important information about meat species identity in raw and processed foods [37] and, in Napoli-salami, contributed to the monitoring of fermentation, processing, storage and safety of final products manufactured with natural extracts instead of nitrates [38]. In food microbiology, the method for separating cell-wall proteins was applied, for example, to several isolates from olive phylloplane and brine previously phenotypically identified as *L.plantarum*, *L. mesenteroides *spp. and *E. faecium*. The high sensitivity of this technique enables discrimination of isolates and distinguishing closely related strains within the same species [39]. Lab-on-chip technology has also been used to detect pathogens such as *E. coli* [40] and other foodborne pathogens [41]; nowadays different commercially available chip-based pathogen sensing systems are available [42]. Lab-on-chip electrophoresis is one of the most promising approaches in the analysis of proteins; however, its main disadvantage remains the impossibility of recovering the proteins, usually admitted by normal gel electrophoresis. On the other hand, such an approach could be considered as complementary to the usual gel-electrophoresis methods, giving us, in a short time, a complete overview of the protein profile present in a certain sample and allowing us to process, if necessary, such a sample by a conventional gel-electrophoresis, and subsequently to recover the protein of interest through use of common extraction procedures from conventional SDS-PAGE. 

## 2. Proteomics-on-a-Chip: Promising Development in Food Science?

*Highlights:* Proteomics may represent a valid means for the identification and evaluation of food quality; “Proteomics-on-chip” in a miniaturized way, allows us to separate in short time, all proteins contained in a biological sample, by isoelectrofocusing and size; Proteomics-on-chip can represent a promising complementary approach to techniques, such as mass spectrometry, in the identification of proteins of interest in food quality and safety.

Proteomics can be considered to be one of the invaluable factors in elucidating the huge complexity of biological processes. Pandey and Mann [43] defined proteomics as “the study of the entire protein complement expressed by a genome in a cell or tissue type”. It can be considered as a fundamental bridge between transcriptomics and metabolomics, acting as an essential counterpart for genomic research through the identification of those corresponding and specific proteins arising from gene transcripts. The abundance of information and results supplied by proteomics can supply a wide spectrum of new tools that can also be used in the field of food science and technology. Proteomics gives the chance to characterize in a new way the protein component of foods: Through the application of gel and non-gel approaches. Such techniques permit the investigation of differences in the protein composition of the tissues of a specific animal or vegetal food type, during growth, maturation, post mortem or post-harvest, and during treatments such as cooking or preservation. In food microbiology, proteomics allows us to monitor and identify the different species involved in food manufacturing, for quality and safety, contributing to detection, monitoring and control of food spoilage, as well as in the investigation of the presence of beneficial or starter microorganisms [44]. For certain sorts of foods, such as wheat, wine, meat and fish, proteomics may represent a valid source for their identification and for the evaluation of their quality [45,46,47,48]. Potential challenges of proteomics in fruit and vegetable research were seen in the identification, for example, of the presence of amino-cyclopropane-carboxylate (ACC) oxidase related to fruit ripening. However, due to technical limitations, only a limited number of proteins were identified in apples and bananas related to their ripening [49,50]. Proteomic approaches, based on 2-DE technology using IGP strips, IEF/SDS-PAGE and mass spectrometry analysis enabled the identification of 22 proteins related to tomato ripening [51] or, by 2-DE and MALDI/TOF, of some proteins expressed after tomato infection with blossom-end rot [52]. The proteome and/or metabolome of starter cultures in fermentation processes of cheese, sausage, beer, *etc.*, can be used to predict the quality of the fermented endproduct [22,53].

**Figure 4:**
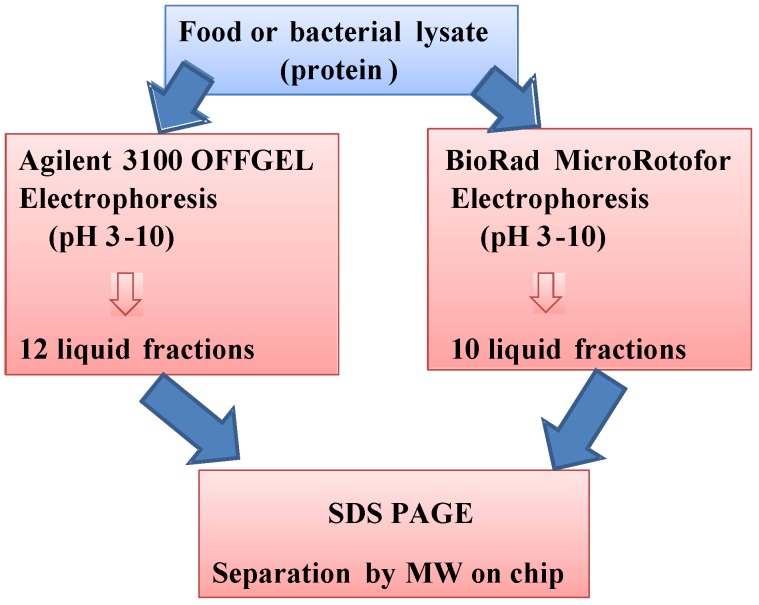
Comparison of the two systems of microelectrophoresis OffGel^®^ (Agilent) and Microrotofor^®^ (Bio-Rad).

Among proteomic techniques, two-dimensional electrophoresis (2-DE), a gel based procedure, is capable of resolving thousands of proteins, so that it is possible to have a precise idea, with a subsequent step represented by mass spectrometry [54], of peptide composition, sequencing and identification. The gel-based 2-DE technique, introduced by O’Farrel in 1975 [55], is based on its capability of separating proteins by their iso-electric point (pI) and size. During the last decades, the performance of this technique has been improved: It has become more reliable with the development of stable immobilized pH gradient (IPG) based systems having high resolving power and improved reproducibility [56]. Although there are exceptional challenges in conventional proteomics procedures, complete proteome profiling comes from a series of steps also requiring several days of laboratory intensive work, suffering from manual operation, long cycle times, and low sample throughput. These factors essentially address the research and technology to enhance the analytical capabilities of proteomics, diminishing the cost, the consumption of materials and reagents, and shortening processing times. One of the most promising improvements can be represented by the so-called “proteomics-on-chip”, which, in a miniaturized way, can separate, by pI and size, all proteins contained in a biological sample. Indeed, the data obtained by the micro-fabricated device is comparable to those obtained by a conventional 2D-PAGE. The prototype 2D chip electrophoresis was realized in 2002 by Chen *et al.* [57]. Generally, 2D gel electrophoresis on chip is performed by a first dimension, carried out in a 1D capillary, with this system physically isolated from the capillaries that provide the separation in the second dimension. After completion of the first separation, a second separation is carried out in an orthogonal set of parallel capillaries, physically connected to that 1D. Currently, the capability of polydimethylsiloxane (PDMS) to support the fabrication of 3D microfluidic systems makes it possible to produce membranes that enclose the gel used in the first separation in a capillary and provides passages for the proteins to migrate into the array of orthogonal capillaries. Another micro-fabricated device, which replaces the conventional 2D-PAGE, comprises a micro-isoelectric focusing (μIEF, 1D) unit that allows the separation of proteins into liquid fractions based on their isoelectric point (pI). Fractionation by liquid-phase IEF is of particular benefit for those proteins that are insoluble or otherwise do not separate well in other, gel-based IEF media. In addition, IEF in solution enables fractionation of proteins in their native state by pI, when it is performed at the appropriate temperature. The technology, on a bigger scale, was used by Bier *et al*. [58], and then used for the liquid fractionation of different kinds of biological matrices, cells or sub-cellular fraction [59,60]. In recent years, the system has been refined, allowing for the use of a small amount of sample. A system developed by Bio-Rad, called MicroRotofor^®^ consents to separate proteins contained in 2.5 mL by a preparative isoelectric focusing (IEF) in a very short time (about 2–3 h). This system uses a cylindrical focusing chamber divided into ten compartments (each containing a single fraction) by nine parallel, monofilament polyester screens. After focusing, the solution in each compartment is collected without mixing using the vacuum-assisted harvesting station that is integrated within the cell [61]. The OFFGEL Fractionator^®^ (Agilent) performs isoelectric focusing of proteins or peptides in immobilized pH gradient (IPG) gel strips. Also in this case, proteins or peptides present in the sample do not remain in the gel. Instead, they are recovered from a buffer solution, making the recovery much easier than with conventional gels. After fractionation, the liquid fractions containing pI-based separated proteins or peptides can easily be removed with a pipette and processed for downstream experiments. The advantageous feature of this instrument is that it can work for low volumes, typically of the order of 0.1 to 1 mL [62]. The two systems are compared in Figure 4.

**Figure 5:**
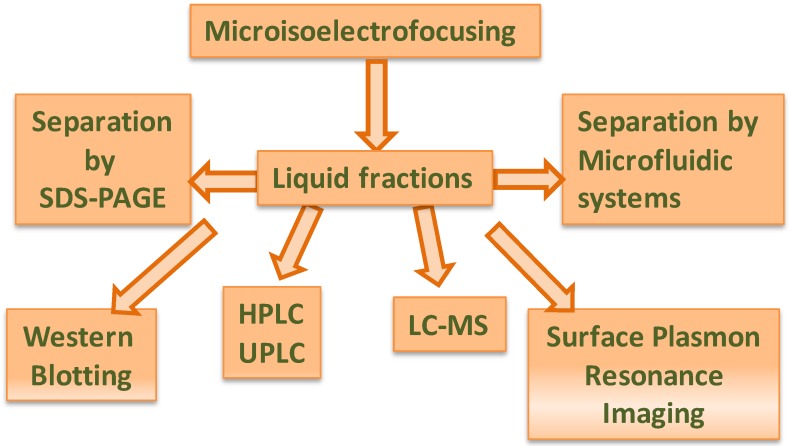
Some examples of analysis after microisoelectrofocusing.

Undoubtedly, proteomics-on-chip cannot be considered to be the most sensitive and resolving method for analysis, mainly for the identification of proteins, compared, for example, to mass spectrometry; however, due to its relative speed of operation, it can be thought of as a complementary step to other more complex and finer analytical approaches, for the isolation and identification of molecular markers of quality and identity in food science. Many downstream applications can be applied after liquid pI fractionation of proteins, such as pooling fractions together for 2D gel electrophoresis of narrow pH range proteins, protein enrichment for Western blot analysis, and HPLC separation for subsequent proteomics analysis. Liquid fractions from IEF separation can also be re-fractionated for further purification, or, when present in their native state, they can be subjected to native electrophoresis, in the case of detection of biological activity, or subjected to a conventional SDS-PAGE (2-DE) or to electrophoresis on microchip (μ2DE), or processed by surface plasmon resonance imaging (3D) and/or mass spectrometry (4D) (Figure 5). 

D’Amici *et al.* [63] used the liquid fractionation, through the MicroRotofor^®^, for the first dimensional separation of native protein complexes based on pI. Individual fractions were then further separated by Blue native-PAGE, giving a 2-dimensional separation. Finally, single protein bands were excised and subjected to denaturing SDS-PAGE to obtain a 3D separation.

These new analytical approaches might play a fundamental role in the proteomics of food and food microbiology. However, at least to our knowledge, there are still few applications in food science and microbiology compared to the challenges [64,65]. The pI liquid fractions of some food protein digests were studied after fractionation with the MicroRotofor^®^. The resulting fractions were analyzed by CE and ESI-TOF mass spectrometer using electrospray tips micro-fabricated in polyimide [66]. Surowiec *et al.* [67] used a proteomic approach to find potential markers for the detection of mechanically recovered chicken meat. Intact proteins were extracted from raw meat and then analyzed with OFF-GEL electrophoresis followed by SDS-PAGE and identification of potential markers by nano-LC-MS/MS. It was shown that it is possible to extract, separate and identify key proteins from processed meat material. 

**Figure 6:**
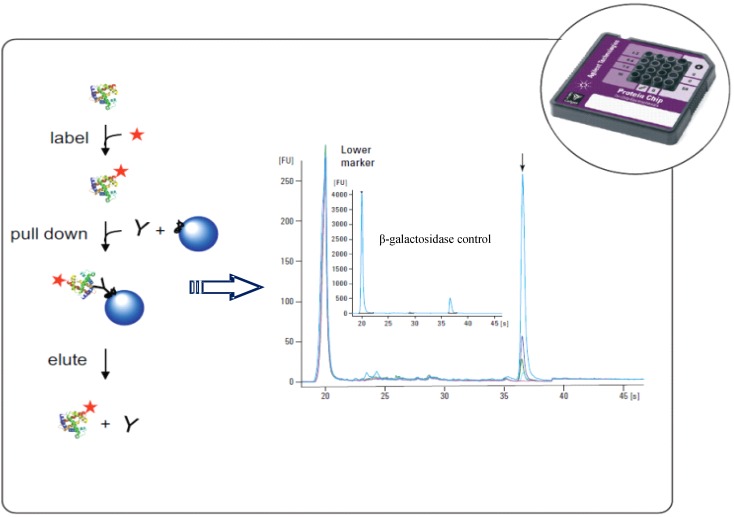
Immunoprecipitation combined with microcapillary electrophoresis. Adapted from [70].

The fractionation with MicroRotofor^®^ system followed by separation by MW on Pro260chip (Experion^®^) was used for the analysis of myofibrillar proteins from different animal species, with the aim of detecting and identifying some molecular markers of quality [68]. More recently, the same methodology was used to evaluate the influence of different carbohydrates used as energy sources on the protein profile of the probiotic *Lactobacillus acidophilus* [69]. Use of such an approach allowed the identification of some protein markers, of different size and pI, present in the whole bacterial proteome when the microorganism was pre-grown in the presence of inulin and pectin. This could play a role in bacterial resistance in simulated gastro-intestinal conditions and for the production of some functional short chain fatty acids, such as butyric acid. Herwig *et al.* [70] developed a method for a sensitive, selective and quantitative detection, of the endogenous β-galactosidase of *E. coli*, the determination and quantification of which is usually based on spectroscopic methods using enzymatic activity (Figure 6). The strategy combined immunoprecipitation with automated SDS-microchip capillary gel electrophoresis (IP-MCGE) using the Agilent 2100 Bioanalyzer^®^, allowing shortening of the cycle time and consumption of less reagents compared to the classical Western blotting; in addition, it could quantify across a concentration range of 625–12500 pg/μL.

## 3. Conclusion

The systems described could support modern research in several areas of food science, including nutrition, origin, authenticity, processing, packaging, quality, safety and food microbiology, due to the interesting possibilities supplied in terms of selectivity, sensitivity and speed of analysis in all areas requiring such discipline. Owing to increasing interest, it is expected that the application of such systems will become routine in the near future.
